# Dry needling has lasting analgesic effect in shoulder pain: a double-blind, sham-controlled trial

**DOI:** 10.1097/PR9.0000000000000939

**Published:** 2021-06-28

**Authors:** Marcus Yu Bin Pai, Juliana Takiguti Toma, Helena Hideko Seguchi Kaziyama, Clarice Listik, Ricardo Galhardoni, Lin Tchia Yeng, Manoel Jacobsen Teixeira, Daniel Ciampi de Andrade

**Affiliations:** a LIM-62, Pain Center, Department of Neurology, University of São Paulo, São Paulo, SP, Brazil; b Division of Physical Medicine and Rehabilitation, Instituto de Ortopedia e Traumatologia, University of São Paulo, São Paulo, SP, Brazil; c Pain Center, Instituto do Câncer Octavio Frias de Oliveira, University of São Paulo, São Paulo, SP, Brazil

**Keywords:** Myofascial pain, Chronic pain, Dry needling, Trigger points, Shoulder pain, Quantitative sensory testing

## Abstract

**Introduction:**

Myofascial pain syndrome (MPS) affects most patients with chronic shoulder pain. Dry needling (DN) is a common treatment for MPS, but its temporal pattern and sensory effects remain unknown.

**Objectives:**

We evaluated in a randomized, sham-controlled study the pattern of analgesic efficacy and local sensory changes of a single session of DN for MPS in patients with chronic shoulder pain.

**Methods:**

Patients with chronic shoulder pain were randomized into active (n = 20) or sham (n = 21) groups. A single DN was performed by a researcher blinded to group assignment and pain outcomes. Pain intensity was assessed by the numeric rating score, and sensory thresholds were evaluated with a quantitative sensory testing protocol, including the area of tactile sensory abnormalities 7 days before needling, right before, and 7 days after the intervention.

**Results:**

Dry needling led to significant larger pain intensity reduction (from 6.30 ± 2.05 to 2.40 ± 2.45 in the active group; *P* = 0.02, effect size = −1.3 (95% CI [−2.0 to −0.68]); (number necessary to treat = 2.1). Pain reduction scores were significantly different on the second day after needling and persisted so until the seventh day and were accompanied by improvement in other dimensions of pain and a decrease in the area of mechanical hyperalgesia in the active DN group alone (*P* < 0.05).

**Conclusion:**

Active trigger points DN provided analgesic effects compared with sham and decreased the area of local mechanical hyperalgesia. These findings have practical clinical implications and may provide mechanistic insights behind MPS.

## Abstract 2

Supplemental Digital Content is Available in the Text.

Dry needling has analgesic effects in shoulder pain associated with myofascial pain syndrome. The analgesic effects last for up to 1 week.

## 1. Introduction

Musculoskeletal pain disorders rank as the 10th leading cause of years lived with disability worldwide.^27^ Shoulder pain is responsible for up to 20% of musculoskeletal complaints,^39,52^ leading to inability to work, loss of productivity, and a considerable burden for the patient and society.^42^ Shoulder pain is a common complaint in all ages, and it is one of the major reasons why patients consult with primary health care providers.^23,42^ The lifetime prevalence of shoulder disorders may affect up to 70% of the population.^8^

Myofascial pain syndrome (MPS) is characterized by local and referred pain because of the occurrence of tenderness in a taut, palpable band of muscle fibers, where painful hyperalgesic myofascial trigger points (MTrP) are identified by manual palpation.^32^ Myofascial trigger points occur due to dysfunctional endplate potential and excessive acetylcholine release in the neuromuscular junction that prevents muscle fibers from fully relaxing. It usually arises from muscle overload secondary to inadequate postures or overuse from repetitive activities or as part of referred pain from deeper injured structures, resulting in increased local tenderness and pain.^9,21,30^

Myofascial pain syndrome is highly prevalent and is considered one of the most common mechanisms behind shoulder disorders, affecting up to 95% of patients.^50^ Myofascial pain syndrome is frequently found in nociceptive shoulder pain and is believed to be the main cause of pain or coexist and contribute to shoulder pain occurring due to other etiologies, such as subacromial impingement syndrome bursitis, and rotator cuff syndrome.^6^ Myofascial pain syndrome is associated with disability and dysfunction because of decreased range of motion of the girdle joints.^6^

A variety of therapeutic techniques have been proposed to treat trigger points and MPS.^37^ Nonpharmacological approaches are widely used^50^ and generally preferred over pharmacological ones because of better tolerance and safer adverse event profiles.^18^ Dry needling (DN) is a minimally invasive procedure, consisting of the use of a fine, solid filiform needle repetitively inserted into the fascia and muscle in a fan-like technique (Video S1, available at http://links.lww.com/PR9/A112; Supplemental Digital Content 1, available at http://links.lww.com/PR9/A111). Techniques analogous to DN have been used for over a century in Western Medicine (see description from Sir William Osler in *Principles and Practice of Medicine* in 1912).^30^ Dry needling is believed to cause musculoskeletal pain relief^32^ and improvement in range of motion by triggering a local twitch response,^1^ subsequently leading to a temporary attenuation or disappearance of MTrPs. The dry needling of MTrPs can result in a mechanical reduction of peripheral nociceptive inputs from the muscles,^9^ contributing to peripheral, spinal, and supraspinal desensitization, along with activation of multiple central pain regulatory pathways,^21^ and functional restoration of neuromyofascial tissues.^9^ Dry needling reduces the irritability of neuromuscular junctions (motor endplate noise)^2^ and sympathetic overactivity in the affected regions, effectively reducing the overlap of the contractile proteins and relaxing the sarcomeres.^48^ Dry needling is usually performed at active MTrPs.^21,37^ It is believed that treatment of the trigger point, and thus removal of the peripheral source of nociceptive stimulus can reduce mechanical hyperalgesia and allodynia, as observed in migraine^31^ and whiplashinjury.^25^ Although needling of MTrPs is part of the daily practice of physicians dedicated to the treatment of musculoskeletal pain, there is still limited clinical evidence for its actual efficacy, as few clinical trials have evaluated its effects in chronic shoulder pain^18,28^ against a proper sham needling^17,38^ and for a sufficient length of time.

The purpose of this study was to evaluate the actual analgesic effects of a DN session on shoulder pain associated with MPS in a double-blind controlled study. We have also explored the concomitant changes in cutaneous sensory thresholds with a battery of quantitative sensory testing (QST) in the area of referred pain triggered caused by DN (eg, secondary hyperalgesia reduction) and its potential role in predicting the temporal persistence of the analgesic effects caused by the needling procedure.

## 2. Methods

### 2.1. Study design

**Figure 1.**
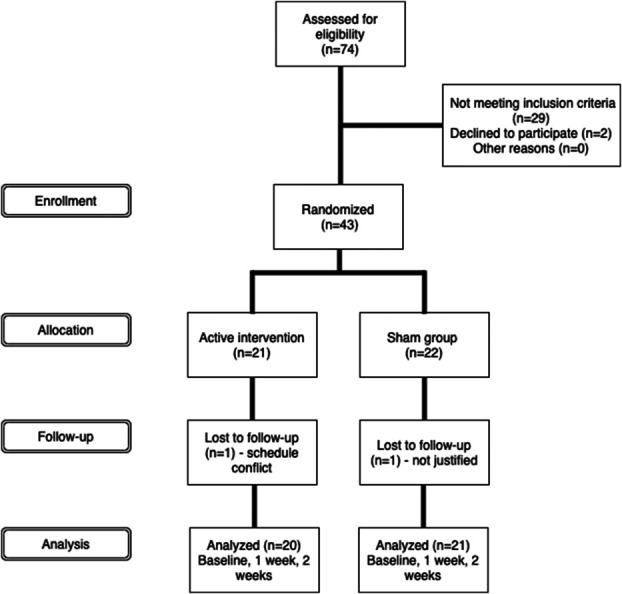
Participant enrollment.

This study was designed as a 2-parallel arm, randomized, and sham-controlled trial, with an allocation ratio of 1:1. The study was approved by our institution's ethics review board (# 0447/10), and all patients provided written informed consent before inclusion in the study. The trial was registered at Clinical Trials (#NCT02179320). Participant enrollment is presented in Figure 1. A total of 74 patients were screened for participation, 43 patients were randomized, 21 for the active and 22 for the sham group.

### 2.2. Patients

Consecutive patients were recruited in several pain clinics in the area of São Paulo, Brazil, and assessed at the Pain Center of the Hospital das Clinicas of the University of Sao Paulo, Brazil. All patients had chronic nociceptive shoulder pain where MPS^50^ was considered to be present and constituted a major cause of pain according to the assessment of 2 independent physiatrists (M.B.P. and J.T.T.). Inclusion criteria included individuals aged 18 to 70 years, presence of chronic unilateral shoulder pain or asymmetrical bilateral shoulder pain, with the most painful side presenting a score of at least 40/100 mm higher in the visual analogue scale (VAS, ranging from 0: no pain to 100: maximal pain imaginable) compared with the less painful shoulder. Other inclusion criteria included the presence of nontraumatic chronic shoulder pain because of at least one of the trapezius muscle trigger points^9^ and pain duration longer than 3 months (>15 days per month with pain). Concomitant medication for pain and sleep disorders was allowed, provided that their doses were stable for at least 30 days before enrollment and remained unchanged during the study. Patients were not included if evidence of neuropathic pain was present (ie, a positive *Douleur Neuropatique*-4), if they had intermittent pain patterns (<15 days per month), if they refused to provide consent for participation, or if they had evidence of another painful shoulder disorder such as subacromial impingement syndrome, adhesive capsulitis, calcific tendonitis of the rotator cuff, and severe rotator cuff tendon alterations. All patients underwent shoulder radiography and, in some instances, ultrasound examinations to exclude major structural disorders. Patients with known fibromyalgia or rheumatic diseases were excluded.^3,54^ Patients with a current primary psychiatric condition, including major depression or major personality disorders according to *Diagnostic and Statistical Manual of Mental Disorders-IV* criteria and a history of drug or alcohol abuse based on the CAGE^40^ questionnaire, were excluded. Patients were also excluded if they were to be enrolled in another clinical trial during the study or if they had participated in a clinical trial within the previous 6 months before enrollment.

### 2.3. Experimental design

Randomization was performed through the website www.randomizer.org. Patients were matched according to age and sex in blocks of 6. The active needling group (A) was composed of participants who underwent one session of standardized trigger point dry needling and by the sham group (S) receiving a standardized sham session of dry needling,^47^ see supplementary video (Video S1, available at http://links.lww.com/PR9/A112; Supplemental Digital Content 1, which demonstrates standardized treatment procedures, available at http://links.lww.com/PR9/A111).

Patients were assessed in 3 face-to-face visits—D0: 1 week before needling, D7: day of needling, and D14: 1-week postneedling follow-up.

D0—at enrollment, patients were assessed for eligibility. If enrolled, they were instructed to fill in a 14-day pain diary in which the worst, average, and lowest daily pain intensities were recorded, using the self-rating eleven-point numerical rating scale (NRS) ranging from 0 (no pain) to 10 (worst pain) from the Brief Pain Inventory (BPI),^15,24^ to establish a baseline pain level before needling. Patients were also instructed to record any adverse events of the therapy during the study period.

D7—patients were randomly assigned into 2 treatment arms (active or sham treatment). They filled in a preprocedure pain and mood assessment battery. Quantitative sensory testing was performed at 3 sites before and right after the needling procedure at the (1) skin area over the painful trapezium, (2) the contralateral mirror area, and (3) a control area on the trunk (dermatomes T6-8 over of the rib cage, at a site with no local or referred pain) (Table S1, Supplemental Digital Content 2, which demonstrates experimental study outline design, available at http://links.lww.com/PR9/A111).

D14—a third QST battery was performed, and the same pain and mood assessment from baseline were filled in.

### 2.4. Description of the needling procedure

Patients were blinded to which treatment they received. Patients underwent either an active or sham trigger point dry needling to the most painful trapezius muscle. The trigger point was previously localized by firm digital pressure through palpation of the trapezius muscle and pressure algometers with a 3 cm^2^ hard foam tip to provide blunt-ended pressure of at least 2 kg/cm^2^ (Wagner Instruments, Greenwich, CT). The identification was based on the operational definition of MTrPs by locating the presence of a palpable taut band and its hypersensitive area and a local pain response because of the palpation of the taut band or reproduction of referred pain (defined as 80% resemblance) in response to local digital compression.^42^ Patients were seated facing a research assistant, with minimal interpersonal interaction, and needling was performed by a specialist facing the patients' back. The researcher performing the needling procedure had no other role in the study or contact with patients except for the few seconds of the needling procedure duration. Each patient was treated only once. The pain specialist who performed the procedures had to certify that both treatments had the same 20-second duration and were similar in the intensity of transprocedural pain elicited, which was controlled by the measurement of pain intensity on a VAS (0-no pain and 100 mm-maximum pain imaginable) every 5 seconds during needling using a chronometer. The patients were asked to use the hand contralateral to the painful trapezius under treatment to score the VAS. The trigger point inactivation on the active group was performed according to the technique standardized by Simons et al., with 0.25 × 40 mm Huanqiu acupuncture needles. Patients who underwent sham treatment had the needle inserted intradermally, superficially, parallel to the skin, without reaching the muscle and its trigger point. The sham needling technique included twisting the needle in a plane parallel to the fascia so that some pain could be elicited from the procedure but without having the needle inserted into the muscle's trigger point (ie, the putative mechanism of action of active needling) (Video S1, available at http://links.lww.com/PR9/A112; Supplemental Digital Content 1, available at http://links.lww.com/PR9/A111).

### 2.5. Main clinical endpoints

The primary outcome was pain intensity: average pain intensity over the last 24 hours, measured with an eleven-point numerical rating scale—NRS (0 = no pain and 10 = maximum pain imaginable)—7 days after the procedure (D14). Baseline average pain intensity was assessed with the average pain of the 7 days before needling (from day 1 until 7 = baseline), on the day of the procedure (D7, before dry needling), and daily on the remaining 7 days (until day 14). The secondary aim was to assess whether the analgesia because of dry needling correlated with acute DN-related alterations in mechanical hyperalgesia area and other sensory variables, such as cold-induced pain, mechanical hyperalgesia, or mechanical hyperesthesia.

### 2.6. Outcome measurements

(1) The VAS is a self-report pain scale, consisting of a horizontal line of 100 mm in length, that is anchored by the ratings “no pain” at the left side (score 0) and “worst pain imaginable” at the right side (score 100).^16^
(2) The numerical rating scale is a self-rating subjective pain measuring scale that measures pain from 0 (no pain) to 10 (worst pain).^22^
(3) The *Douleur Neuropatique*-4 (DN4) questionnaire used for the screening of neuropathic pain.^4,45^
(4) The BPI allows patients to rate the intensity of their pain and pain interference with daily activities.^15,24^
(5) The short-form McGill Pain Questionnaire consists of 15 descriptors, which evaluate sensory, affective, and cognitive aspects of pain.^41^
(6) The Hospital Anxiety and Depression Scale, a self-assessment scale, was used to evaluate the treatment effects on mood and anxiety.^55^

The global impressions of change consist of a Likert scale with 7 points ranging from “very much improved” to “very much worse” based on the degree perception of change after treatment experienced by the patient and the rater (clinician).^22^ Patients were classified as “improved” or “not improved” with improvement being a significant or moderate improvement and “not improved” being any other score.

### 2.7. Quantitative sensory testing

All participants underwent a QST battery over the painful referred pain on the shoulder, a contralateral mirror area, and an area located ipsilateral to the pain side, over the T6-7 dermatomes over the flank. Quantitative sensory testing changes were compared between sessions at the painful side. The QST battery assessed large fiber (Aβ) and small (Aδ and C) mediated somatic sensory inputs, assessed at 3 time points: before DN, immediately after DN, and on D14 (7 days after the procedure). Evaluation of mechanical static tactile sensitivity was performed with calibrated von Frey monofilaments ranging from 0.008 to 300 g (Senselab Aesthesiometer; Somedic, Sweden), of increasing thicknesses, for determining the threshold of tactile and pain detection, exerting greater pressure on the skin as the monofilament caliber increased. The detection of pain thresholds, supraliminal stimulations with strands 2 and 3 times thicker than the ones used for determining pain threshold was made so that mechanical hyperalgesia was evaluated through the VAS after each stimulus. Finally, the mechanical hyperalgesia area (cm^2^) boundaries were determined with suitable von Frey filaments^43^ and marked using a proper nontoxic pen. This area was then copied through transparent paper, scanned, and digitally quantified in the computer with Adobe Photoshop CS4 11.0. For thermal nonpainful perception and cold hyperalgesia, a custom-made contact thermode (USP, 2016) was applied over the painful trapezius muscle at 2 constant fixed temperatures of 15 and 5°C for 5 seconds.

### 2.8. Safety

The safety of dry needling was assessed by monitoring the occurrence of adverse effects during treatment by a dedicated recording file.

### 2.9. Blinding assessment

The blinding assessment was evaluated with a 4-question form, which asked patients whether they knew which group they were, which intervention they received, their pain intensity during needling, and if they would accept receiving the same treatment again if proposed and their justification.^44^

### 2.10. Statistical analyses

Statistical analyses were conducted with SPSS version 22 (SPSS Inc, Chicago, IL). The categorical data were expressed in proportions, and continuous variables were expressed in mean and standard deviation. The exploratory analysis initially evaluated distributions, frequencies, and percentages for each of the numeric and categorical variables. We assessed randomization effectiveness by evaluating balance regarding baseline variables, comparing the interventional and the control arms. Normality of the data was accessed by the Kolmogorov–Smirnoff test. In all cases, *P* values <0.05 were considered significant. The repeated-measures analysis of variance test was used for the comparison of the outcomes between the groups along the trial, including an interaction term between group and time and post hoc analyses when indicated. Correlation analyses between the main outcome results were performed to verify the association between pain improvement and QST parameters. Only correlations with coefficients above 0.4 were reported. Because the Kolmogorov–Smirnov test revealed that secondary outcomes such as quality of life and QST values did not have a normal distribution, the differences between groups were compared using nonparametric test (Kruskal–Wallis test), followed by pairwise comparisons of change between groups (Wilcoxon/s/Mann–Whitney U-test). Bonferroni correction for multiple comparisons was used in these settings. The sample size was calculated based on the effect size achieved by a previous trial,^51^ considering a repeated-measures analysis of variance approach and using the software G*Power 3.1.9.2 for Windows (California). Bearing in mind the assumptions of an effect size of 0.4 (equivalent to an eta squared effect size of 0.140), 2-tailed *α* error level probability of 0.05, and a minimum power of 0.80, the estimated sample size needed would be 20 subjects per arm. We included 3 extra participants to account for loss of follow-up. Cohen's d, defined as the difference between the means of the 2 groups divided by the pool standard error, was used for the calculation of effect sizes.

## 3. Results

### 3.1. Patients

Data collection took place between February 2015 and January 2016. Two patients were lost during follow-up, one from each group. The reasons for dropping out were specified in Figure 1. Table S2 (Supplemental Digital Content 3, available at http://links.lww.com/PR9/A111) shows the baseline characteristics of the trial participants. There were no significant differences between treatment groups regarding demographic and pain characteristics at baseline (all *P* values >0.2). Patients included in this study had an average age of 58, and most were women (82%). All patients were trigger point dry needling naïve.

### 3.2. Efficacy of dry needling on main outcomes

Dry needling had a significant effect on average pain intensity throughout the treatment, as shown by comparison with the sham group (Table S3, Supplemental Digital Content 4; Figure S1, Supplemental Digital Content 6, available at http://links.lww.com/PR9/A111). The group treated with active needling had significantly lower pain scores than the sham group at follow-up with an average pain intensity change from 6.30 ± 2.05 before the therapy to 2.40 ± 2.46 at the end of treatment (D14) in the active group and 6.04 ± 1.32 before the treatment to 5.14 ± 1.49 at the end of therapy (D14) in the sham group (F(1,39) = 5.908; *P* = 0.02; 95% CI, 1.25 to 3.55, Cohen's d effect size = 1.34 (Cohen, 1988)).

Post hoc analysis with adjustment for multiple testing revealed that the NRS pain score was statistically significantly decreased from baseline to D14 (2.350 [95% CI, 1.781–2.919], *P* < 0.001). There was also a statistically significant difference in NRS at D14 between groups, F (1, 39) = 74.41, *P* < 0.01, partial η2 = 0.317. There was a statistically significant effect of time on NRS pain for the sham group, F (1, 20) = 7.211, *P* < 0.014, partial η2 = 0.265, and for the active DN group, F (1,19) = 55.682, *P* < 0.001, partial η2 = 0.746 (Table S4, Supplemental Digital Content 5, available at http://links.lww.com/PR9/A111).

### 3.3. Efficacy of dry needling and its immediate effects on pain

One single session of dry needling resulted in significant pain reduction in the BPI-worst and BPI*-*average pain (Figure S2, Supplemental Digital Content 7, available at http://links.lww.com/PR9/A111) score starting from D9 (2 days after needling) until D14 (Table 1), suggesting a sustained persistent analgesic effect in the active group only during this period. There was a statistically significant interaction between the intervention and time on BPI-average pain reduction from D9 to D14, F (7273) = 3.047, *P* = 0.004, 95% CI, 0.565 to 3.174, and BPI-worst pain reduction from D9 to D14, F (7273) = 2.959, *P* = 0.005, 95% CI 0.591 to 3.223. We found no significant pain reduction for the weakest pain in any of the evaluated days.

**Table 1:** Results of the effects of dry needling on pain.

	Baseline	D8	D9	D10	D11	D12	D13	D14
BPI-worst pain active	6.84 ± 1.58	5.50 ± 3.03	4.65 ± 2.71*	4.50 ± 2.46*	4.65 ± 2.73*	4.25 ± 2.76*	4.05 ± 2.41*	3.95 ± 2.25*
BPI-worst pain sham	7.50 ± 1.33	6.80 ± 2.29	6.66 ± 2.61	6.57 ± 2.18	6.57 ± 2.29	6.23 ± 2.23	6.42 ± 2.71	6.85 ± 2.03
BPI-average pain active	5.11 ± 1.81	4.20 ± 2.82	3.40 ± 2.43*	3.10 ± 1.99*	3.55 ± 2.52*	3.15 ± 2.43*	2.90 ± 1.97*	2.70 ± 1.89*
BPI-average pain sham	5.84 ± 1.76	5.04 ± 2.41	5.23 ± 2.50	5.28 ± 2.72	5.23 ± 2.91	4.90 ± 2.46	5.04 ± 3.02	5.42 ± 2.22
BPI-lowest pain active	3.75 ± 1.68	3.20 ± 2.70	2.60 ± 2.08	2.70 ± 1.89	2.65 ± 2.05	2.45 ± 2.18	2.30 ± 19.2	1.90 ± 1.74
BPI-lowest pain sham	4.35 ± 2.11	4.09 ± 2.32	4.14 ± 2.43	4.00 ± 2.68	4.28 ± 2.41	4.04 ± 2.39	4.14 ± 2.63	5.42 ± 2.22

Values are presented as mean ± standard deviation.

* *P* < 0.05.

BPI, Brief Pain Inventory.

### 3.4. Effects of dry needling on pain secondary outcomes

Active dry needling significantly improved the BPI-pain interference score, with patients reporting a marked decrease in the interference of pain with “general activities,” “mood,” and “sleep,” compared with the sham procedure. Dry needling had a significant effect on MPQ evaluative dimension of pain, but not on affective or sensory ones. Mean anxiety and depression scores measured on the Hospital Anxiety and Depression scale were not significantly affected by DN (Table 2). Patients in the active group reported 80.0% and 75.0% of “much improvement” in global impression of change—patient and clinician versions, respectively—compared with 33.3% and 42.9% for the sham group (*P* = 0.030 and *P* = 0.037; respectively), the number necessary to treat = 2.1.

**Table 2:** Results of secondary assessments.

	Baseline (D0)		Effect 1 wk after needling (D14)		*P*	Effect size
	Active	Sham	Active	Sham		
HAD depression	6.55 ± 4.65	7.71 ± 3.67	6.80 ± 3.92	7.66 ± 3.38	0.659	0.005
HAD anxiety	8.25 ± 3.76	10.1 ± 2.71	7.75 ± 3.02	9.61 ± 2.81	0.869	0.001
McGill VAS	6.30 ± 2.05*	6.57 ± 1.74	2.40 ± 2.45*	5.42 ± 1.71	**<0.001**	0.363
McGill sensory	4.40 ± 1.63	4.66 ± 1.52	3.90 ± 2.24	5.00 ± 2.28	0.226	0.037
McGill affective	3.25 ± 1.55	3.57 ± 1.24	2.55 ± 1.76	2.85 ± 1.52	0.979	0.0001
McGill evaluative	1.45 ± 0.51	1.42 ± 0.50	1.00 ± 0.64	1.33 ± 0.65	**0.011**	0.064
McGill 3 total dimensions	9.19 ± 3.84	9.66 ± 2.26	7.45 ± 4.37	9.19 ± 3.84	0.346	0.023
BPI %24 h	40.75 ± 33.01	45.23 ± 32.49	60.00 ± 31.95	45.23 ± 30.59	0.160	0.050
BPI worst pain	7.25 ± 2.48	7.57 ± 2.35	4.15 ± 2.60	6.28 ± 3.31	**0.035**	0.110
BPI average pain	4.50 ± 2.13	5.33 ± 2.37	2.55 ± 2.25	4.47 ± 3.09	0.090	0.072
BPI lowest pain	2.65 ± 1.95	3.80 ± 2.74	1.95 ± 2.06	3.14 ± 2.81	0.948	0.000
BPI current pain	4.55 ± 2.81	5.42 ± 3.29	2.80 ± 2.74	4.23 ± 2.89	0.510	0.011
BPI general activities	5.50 ± 3.88	4.95 ± 3.13	2.20 ± 2.62	4.66 ± 3.18	**0.002**	0.229
BPI mood	4.80 ± 3.45	4.61 ± 3.66	2.80 ± 2.21	4.38 ± 3.76	**0.037**	0.107
BPI work	4.25 ± 3.55	5.38 ± 4.21	2.95 ± 2.87	4.14 ± 3.67	0.948	0.000
BPI relationships	1.40 ± 3.16	2.38 ± 4.09	1.50 ± 3.15	2.80 ± 3.85	0.746	0.003
BPI sleep	4.65 ± 3.51	5.57 ± 3.10	2.90 ± 3.29	5.47 ± 3.58	**0.020**	0.131
BPI enjoyment of life	2.30 ± 3.65	4.42 ± 3.94	1.45 ± 2.52	4.28 ± 4.08	0.526	0.010
BPI walk	0.65 ± 1.42	1.85 ± 3.16	0.55 ± 1.82	1.57 ± 3.02	0.868	0.001
BPI total interference (sum)	23.55 ± 14.37	29.19 ± 17.73	14.35 ± 11.93	27.33 ± 19.12	**0.037**	0.106

* *P*, 0.05. Bold indicates statistically significant group effect. Comparison of the effects of dry needling or sham stimulation, from day 7 to day 14, on the HAD depression and anxiety scores, the McGill questionnaire sensory, affective and evaluative scores, BPI total interference score, and its effect size. Data presented as mean ± standard deviation. *P*-value for the interaction between group and time.

BPI, Brief Pain Inventory; HAD, Hospital Anxiety and depression scale.

### 3.5. Effects of dry needling on quantitative sensory testing

Dry needling produced a significant reduction in mechanical hyperalgesia on the skin over the painful area after needling (49.2 ± 37.4 cm^2^ at baseline [D7], 39.2 ± 42.7 immediately after needling on D7, and 30.3 ± 28.5 cm^2^ on D14, *P* = 0.001), for the active group when compared with sham stimulation. Other QST variables were not affected by treatment (Table S5, Supplemental Digital Content 5, available at http://links.lww.com/PR9/A111).

### 3.6. Medication use

Patients had an average MQS of 7.50 ± 3.18 in active and 7.14 ± 3.19 in sham groups at baseline (*P* = 0.67). At D14, MQS for the sham group was 6.85 ± 3.08 (*P* = 0.55) and 7.10 ± 3.07 (*P* = 0.57) in the active groups.

### 3.7. Correlation analyses

As expected, improvement of pain intensity was significantly correlated with an improvement on global impression of change both for patients and clinicians (rho = −0.630, *P* = 0.003, and rho = −0.630, *P* = 0.003, respectively). There was no correlation between BPI-average pain intensity improvement and changes on the area of mechanical hyperalgesia. Interestingly, we found a correlation between daily pain improvement starting 2 days (Figure S3, Supplemental Digital Content 8, available at http://links.lww.com/PR9/A111) after active dry needling and a higher pain reduction during the following days until the last assessment (D10: rho = 0.590, *P* = 0.013; D11: rho = 0.512, *P* = 0.21; D12: rho = 0.772, *P* = 0.0001; D13: rho = 0.752, *P* = 0.0001; and D14: rho = 0.670, *P* = 0.001).

Also, patients who presented an immediate mechanical hyperalgesia area reduction after needling had a positive correlation with maintaining this positive area reduction response after 7 days on D14 (rho = 0.436, *P* = 0.004) (Figure S4, Supplemental Digital Content 9, available at http://links.lww.com/PR9/A111). In addition, patients who had a reduction of the area of mechanical hyperalgesia area had a positive correlation with decreasing mechanical pain threshold at D14 (rho = 0.413, *P* = 0.007) (Figure S5, Supplemental Digital Content 10, available at http://links.lww.com/PR9/A111).

### 3.8. Adverse events

The dry needling treatment was well tolerated by patients. No major adverse events were reported from any patient included in this trial. Minor side effects such as minor local pain after dry needling were reported by 4 patients in the active group and 3 patients in sham group, with no functional impact.

### 3.9. Blinding assessment

At the end of the study, 45% of the participants in the active group reported they were able to tell in which group they were allocated to, and among them, 55% guessed it right. In the sham group, these proportions were 62% and 47%, respectively. When asked if the patients would like to maintain active dry needling sessions for a longer period, should this option be offered to them, affirmative answers were given by 70% of the active group and 55% of the placebo group. These proportions were not significantly different.

## 4. Discussion

We have shown that patients with chronic shoulder pain treated by dry needling had a significant improvement in pain intensity and pain interference with daily activities compared with sham procedure, an effect that persisted for at least 7 days. Improvements started 2 days after needling and persisted for at least 7 days thereafter. We have also described the temporal pattern of pain relief caused by DN, which started on the second and persisted until the seventh day postprocedure. The study also evaluated in a sham-controlled trial the effects of a single session of dry needling on pain intensity and explored the concomitant changes in cutaneous sensory thresholds with a battery of QST in the area of referred pain (eg, secondary hyperalgesia reduction) and its potential role in predicting the temporal persistence of the analgesic effects caused by the needle procedure.

DN analgesic effects were not limited to pain intensity, but also included positive effects of DN on pain interference with daily activities and improvement in global impressions of change. These are original information that add to a literature populated by studies devoid of sham arms^6,19,29^ or providing a superficial report on the sham technique^20^ such as its actual procedure, its duration, depth of needle insertion,^20^ or pain intensity triggered by the sham procedure.^13,51^ This last point is of significant importance because pain during sham needling may, by itself, engage nonspecific top–down pain modulatory systems and trigger pain relief that would be not specific and not related to the trigger point treatment per se, being simply the fruit of the pain suppressive effect of a stronger concomitant nociceptive stimulus.^46^ Here, we took special care to control for the duration and for the intensity of both the active DN and its sham version so that the effects of these biases would be mitigated.

Interestingly, the analgesic effects of dry needling were not immediate as would have been expected in the case where its main mechanisms of action would uniquely rely on trigger point deactivation. Contrarily, our findings showed that a rather delayed response took place, commencing 2 days after the procedure, and with a positive correlation between daily pain improvement at this time point and a more pronounced pain reduction at the seventh day postprocedure. Many of the previous studies in the DN literature reported only immediate effects^34,35^ of treatment, which have provided mechanistic insights into the technique in one hand, but limited clinical impact on the other. In addition, this temporal profile of analgesia installation after DN may explain some negative results based on immediate pain assessment after the procedure.^36^ Considering these findings, we hypothesized that clinically meaningful pain improvement occurs after a delay of a few days after dry needling, and it may not be detected acutely. In this line, DN has previously been reported to possess analgesic effects for painful syndromes where myofascial pain was not present, suggesting that DN analgesic effects would rely not only on the mechanical effects of needle insertion and trigger point treatment, but, instead, on the engagement of other pain suppressive mechanisms. For instance, a Cochrane systematic review of 35 randomized controlled trials evaluated the efficacy of dry needling and treatment of mechanical nonspecific low-back pain, with positive evidence of an immediate and short-term pain relief, although with a small effect size.^26^ Similar findings have been reported for nonspecific shoulder pain^11^ lateral epicondylitis-related pain.^53^ In fact, it has been reported that DN targeting MTrPs or the adjacent muscle outside the MTrP area have similar results similar results to those obtained after direct trigger point needling in ... in poststroke shoulder pain.^33^ Mechanisms of pain reduction after DN may involve both local (peripheral)^9^ and central effects.^46^ The local twitch response and mechanical inactivation of the trigger point may result in muscle soreness after the procedure,^50^ which is detected on the day after needling. Trigger point dry needling results in local muscle microtrauma and may disrupt dysfunctional endplates^29^ causing an involuntary spinal cord reflex contraction of the muscle fibers in a taut band (local twitch response), clearing the excessive buildup of acetylcholine.^10^ Although the acute effects on DN over the MTrPs can be immediate, the biochemical changes responsible for the specific effects of needling^48^ compared with shallow treatment by the sham procedure may take hours to days to build up. Some trials have found that deep dry needling is associated with clinically meaningful results for pain and functionality in the short-term with a single session of active and latent MTrP DN^11^ and at 6 months follow-up after up to 4 sessions of DN.^12^

Growing evidence suggests that deep muscle DN per se, irrespective of its effects in MTrPs, may also decrease pain. Indeed, our results further suggested a different main mechanism driving the analgesic effects of dry needling in pain MPS relief, because the main effects occurred after 2 days of the procedure, which would not be expected it treatment of the trigger point were the sole and main responsible for its analgesic effects. We hypothesized that DN might trigger conditioned pain modulation responses, inducing analgesia by descending inhibition. Alternatively, DN may modulate pain by reducing substance P and CGRP concentrations and increasing the release of endogenous opiates, such as beta-endorphin, enkephalin, and dynorphin in nociceptive pathways, causing a decrease in hyperalgesia that would buildup in days.^10^ Also, it has also been suggested that acupuncture (and possibly DN) may engage serotoninergic descending pain inhibitory pathways,^46^ with effects of needling in the release of neuropeptides on serotoninergic neurons because of activation of enkephalin interneurons^14^ that could not take place immediately after needling.

The dry needling procedure is very similar to the ancient “ashi” point acupuncture technique, where an acupuncture needle is inserted into the painful area, irrespective of the presence of MPS or trigger points locally. Early Chinese physicians proposed that targeting painful areas leads to a reduction in muscle tenderness. Our QST results further support the idea of the DN effect dissociated from the acute effects on MTrPs. Dry needling led to a significant reduction in mechanical hyperalgesia area over the painful area right after needling, which also persisted until the seventh day of follow-up. These suppressive effects in secondary hyperalgesia over referred pain area were expected and were already reported. However, these changes did not correlate with clinical pain relief. These data further suggest that acute DN effects on MTrPs and secondary hyperalgesia were independent of the procedure's long-term clinical analgesic properties. Although previous studies have suggested that DN effects in sensory thresholds would correlate with pain relief,^35^ these reports were not based on a broader QST assessment. We believe our findings were due to the use of 2 control areas for QST in this study: the contralateral mirror area over the contralateral shoulder and an ipsilateral area over the trunk. We undertook the 2-control area approach based on the finding that shoulder pain is bilateral in at least 41% of patients,^7^ and this would bias a solely contralateral assessment of QST abnormalities. This methodological choice probably reduced local sensory changes occurring with time and provided a more adapted and specific control area.

Considering the importance of blinding in clinical research, and that dry needling is an interventional treatment, adequate participant blinding has been challenging in interventional trials.^13^ A systematic review evaluated 19 randomized controlled trials of high quality on dry needling in MSK pain in general. Only 10 (52%) included a sham intervention, and only 3 of them actually assessed the quality of blinding.^5^ Our blinding assessment demonstrated that patients could not accurately tell which treatment group they were allocated into, indicating an adequate blinding. To the best of our knowledge, this was the first study to standardize, describe in detail, control for pain during the sham and active procedure, and film the needling intervention, which, we believe, was a major positive methodological improvement.

Our study had some limitations that should be considered in interpreting these results. The treatment of chronic MPS usually requires a course of treatment and not only one single intervention.^50^ Also, because we stopped our assessment on the seventh day after needing, we do not know the analgesic effect's exact time duration. In addition, dry needling is rarely used as a monotherapy in clinical practice, and its effect in multimodal real-life treatment approaches remains to be determined. This randomized controlled trial demonstrated analgesic effects of local dry needling in shoulder pain for patients with chronic shoulder pain because of MPS. Our results suggest a pragmatic next step in trials on DN for pain. Because the analgesic effects persisted for at least 7 days after the procedure, this may impact the dosing of next studies proposing DN as a long-term treatment approach for MPS. One could propose that weekly DN sessions should be used instead of daily session protocols that are costly and decrease treatment compliance.

## Disclosures

The authors have no conflicts of interest to declare.

This study was supported by a research grant from the São Paulo Research Foundation (FAPESP) (academic research initiation scholarship—M. Y. B. Pai and J. T. Toma) and National Council for Scientific and Technological Development (CNPq) (scientific production scholarship—M. J. Teixeira and D. Ciampi de Andrade).

## Supplementary Material

SUPPLEMENTARY MATERIAL

## Appendix A. Supplemental digital content

Supplemental digital content associated with this article can be found online at http://links.lww.com/PR9/A111, http://links.lww.com/PR9/A112.
