# Supplementary material for: Dry needling has lasting analgesic effect in shoulder pain: a double-blind, sham-controlled trial
Source: Pain Rep. 2021 Jun 28;6(2):e939. doi: 10.1097/PR9.0000000000000939 (PMC8240781; doi:10.1097/PR9.0000000000000939)
Supplement: SUPPLEMENTARY MATERIAL [file painreports-6-e939-s001.pdf]

Supplementary material

Video S1. Supplemental Digital Content 1

Video S1

Legend: active and sham needling procedural. The video was a performed with co-authors and volunteers and did not include actual patients.

[Link to Video]

Table S1. Supplemental Digital Content 2

Study design

Visit	Screening	Baseline (D0)	D7	D14
Inclusion / Exclusion criteria	X			
Consent form	X			
MQS	X	X	X	X
Clinical scales				
DN4	X			
NRS	X	X	X	X
Trigger point Evaluation	X	X	X	x
Pain diary		X	X	X
BPI			X	X
McGill			X	X
HAD			X	X
CGI				X
Sensorial testing				
QST			X	X

Legend: Experimental study outline design. Each subject was evaluated on 3 days (D0, D7 and D14). Treatment was performed on D7. QST was performed before and after treatment on D7, and on D14. Pain questionnaires were performed on D7 and D14.MQS = Medication quantification scale, DN4 = Douleur neuropatique 4, NRS= Numeric rating scale, BPI = Brief pain inventory, McGill = McGill short form pain questionnaire, HAD = Hospital anxiety and depression scale, CGI = Clinical global impression scale, QST = Quantitative sensory testing

Table S2. Supplemental Digital Content 3: Demographics and baseline characteristics of patients.

**Characteristics**	**Active**	**Sham**	**p-value**
Age (years)	58.4 ± 14.5	58.2 ± 11.0	0.968
Sex, female n (%)	18 (90%)	16 (76%)	0.228
Pain intensity	6.3 ± 2.0	6.6 ± 1.7	0.651
DN4	2.4	2.4	
**Concomitant treatment**			
Regular analgesics n (%)	11 (55)	9 (42)	0.671
Antidepressants n (%)	3 (15)	3 (14)	0.343
Anticonvulsants n (%)	3 (15)	4 (19)	0.443

Legend: DN4: douleur neuropathique 4 questionnaire. * P<0.05.

Table S3. Supplemental Digital Content 4

Pain intensity scores for the two groups

	NRS (0-10)		
**Variable**	**Baseline**	**D14**	**P**
**Sham**	6.0 ± 1.32	5.1 ± 1.49	0.214
**Active**	6.3 ± 2.05	2.4 ± 2.45	0.040
**P**	0.246	<0.001	0.020[a]

Legend: The values are presented as mean ± standard deviation. NRS: numerical rating scale, BPI: brief pain inventory.[a] p value for the interaction term between group and time (0-10).

Table S4. Supplemental Digital Content 5

Descriptive statistics of pain intensity at baseline and D14

Treatment	Time	Mean	Std. Error	95% Confidence Interval	
				Lower Bound	Upper Bound
Sham DN	Baseline	5.8	0.321	5.128	6.472
	D14	5.0	0.332	4.304	5.696
Active DN	Baseline	6.3	0.459	5.338	7.262
	D14	2.4	0.550	1.250	3.550

Legend: 95% Confidence Interval at D14. DN = dry needling.

Table S5. Supplemental Digital Content 6

Quantitative sensory testing results of dry needling, before, immediately after, and 7 days after dry needling.

Variable	Group	time			p value [a]
		**Before needling (D7)**	**Immediately after needling (D7)**	**D14**	
Area of mechanical hyperalgesia (cm^2)	Active	49.2 ± 37.4	39.2 ± 42.7	30.3 ± 28.5	**0.001[a]**
	Sham	49.3 ± 33.4	44.5 ± 35.7	44.1 ± 34.8	
Mechanical detection threshold (g)	Active	3.0 ± 1.4	2.5 ± 0.7	2.5 ± 0.8	0.419
	Sham	2.7 ± 0.7	2.5 ± 0.7	2.7 ± 1.1	
Mechanical pain threshold (g)	Active	19.1 ± 10.5	16.7 ± 7.9	15.9 ± 8.8	0.712
	Sham	15.9 ± 10.4	15.3 ± 8.1	14.7 ± 7.1	
Mechanical hyperalgesia (VAS 0-100)	Active	30.4 ± 19.0	33.8 ± 25.7	25.0 ± 20.8	0.191
	Sham	28.8 ± 16.5	32.4 ± 20.2	32.1 ± 17.7	
Hyperalgesia (VAS 0-100)	Active	42.6 ± 26.1	39.9 ± 27.1	32.0 ± 24.2	0.200
	Sham	42.0 ± 22.0	42.8 ± 23.1	42.0 ± 18.7	
Cold hyperalgesia (VAS 0-100)	Active	17.9 ± 15.1	18.3 ± 21.6	15.2 ± 17.2	0.897
	Sham	19.4 ± 15.0	21.7 ± 16.9	17.1 ± 11.2	

Legend: VAS: visual analogue scale. Data is presented as mean ± standard deviation.[a] P value for the interaction term between group and time.

Figure S1. Supplemental Digital Content 6

Mean changes in average pain intensity.

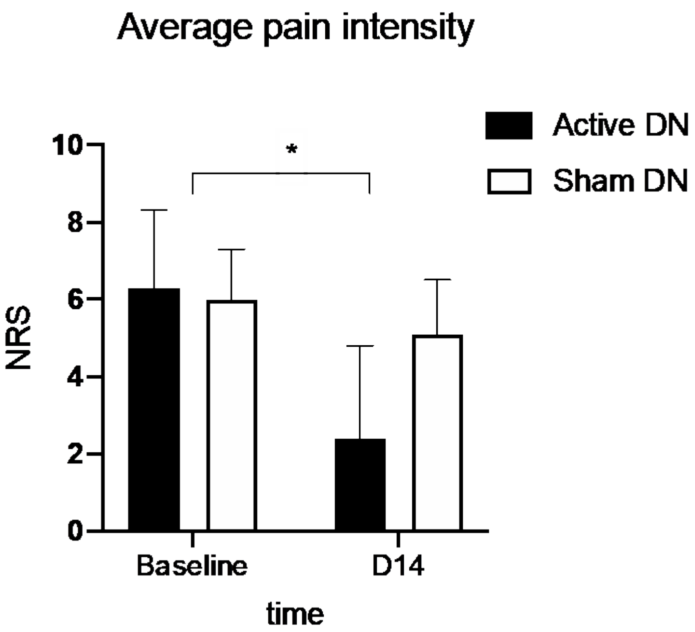

Legend: Error bars represent standard error of the mean (S.E.M.)

Figure S2. Supplemental Digital Content 7

Changes in pain intensity for average and worst pain over time.

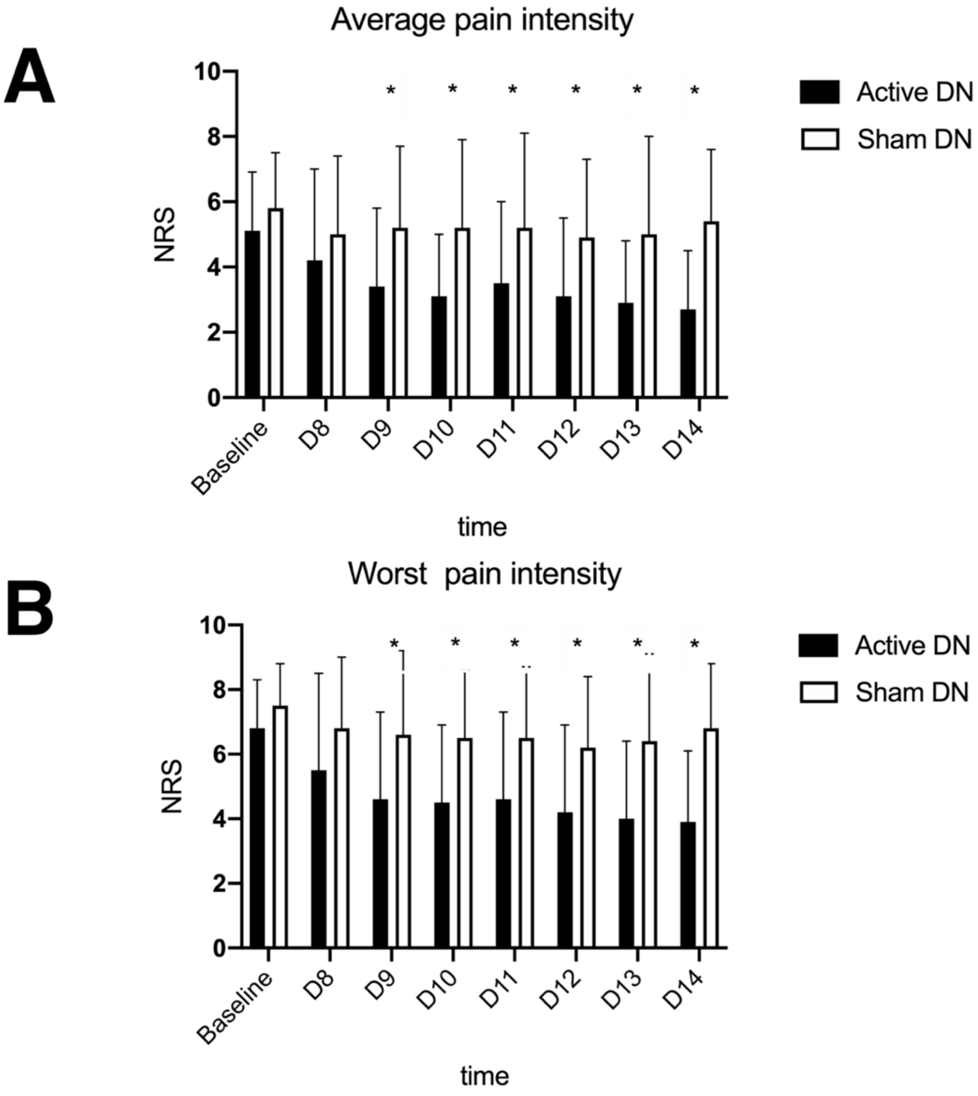

Legend: Error bars represent standard error of the mean (S.E.M.). *P<0.05

Figure S3. Supplemental Digital Content 8

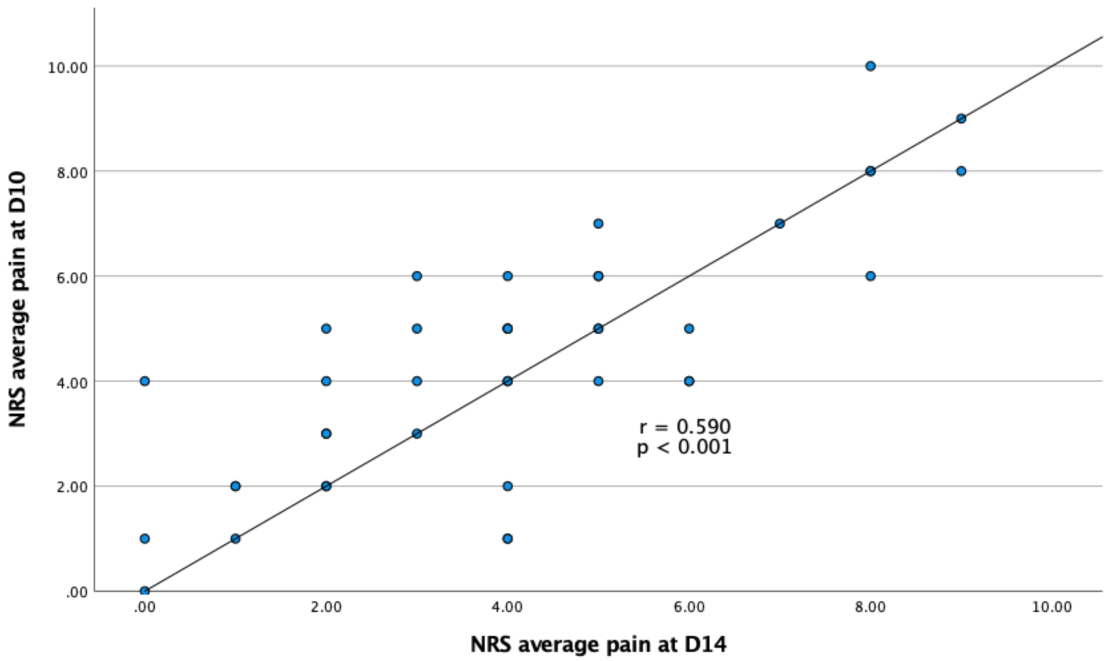

Scatterplot of NRS average pain at D10 versus NRS average pain at D14. The regression line shows an average positive correlation (r = 0.590, p < 0.001), indicating that pain improvement in D9 is correlated with persistent pain improvement at D14.

Figure S4. Supplemental Digital Content 9

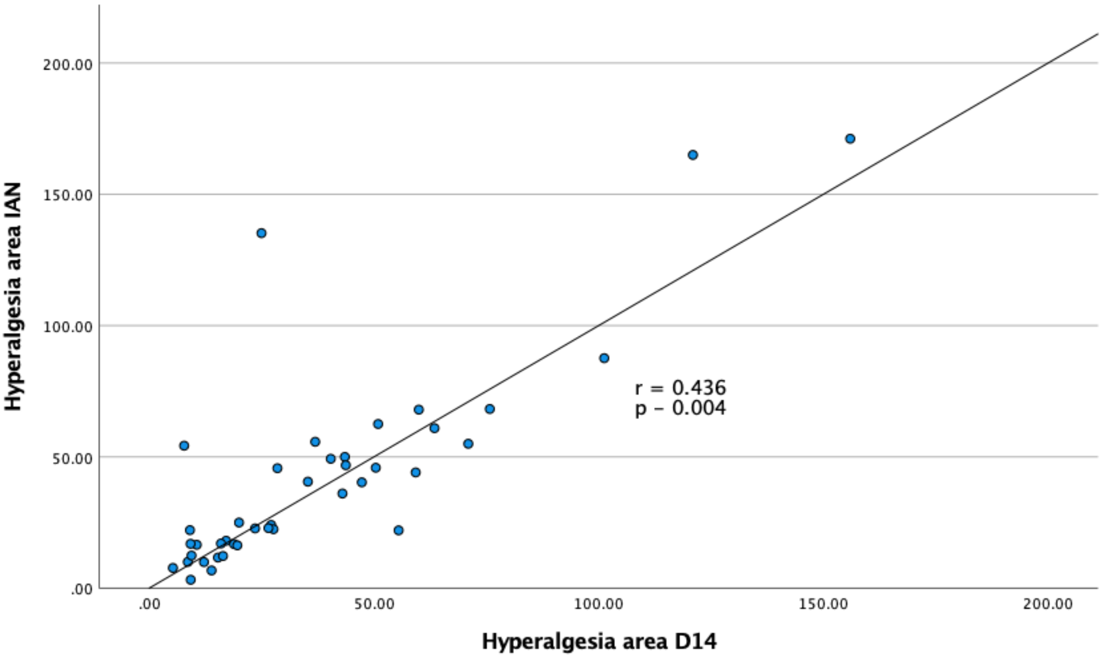

Scatterplot of immediate mechanical hyperalgesia area reduction after needling (IAN) versus mechanical hyperalgesia at D14. The regression line shows an average positive correlation (r = 0.436, p = 0.004), indicating that immediate reduction after needling is correlated with maintenance of this positive area reduction response at D14.

Figure S5. Supplemental Digital Content 10

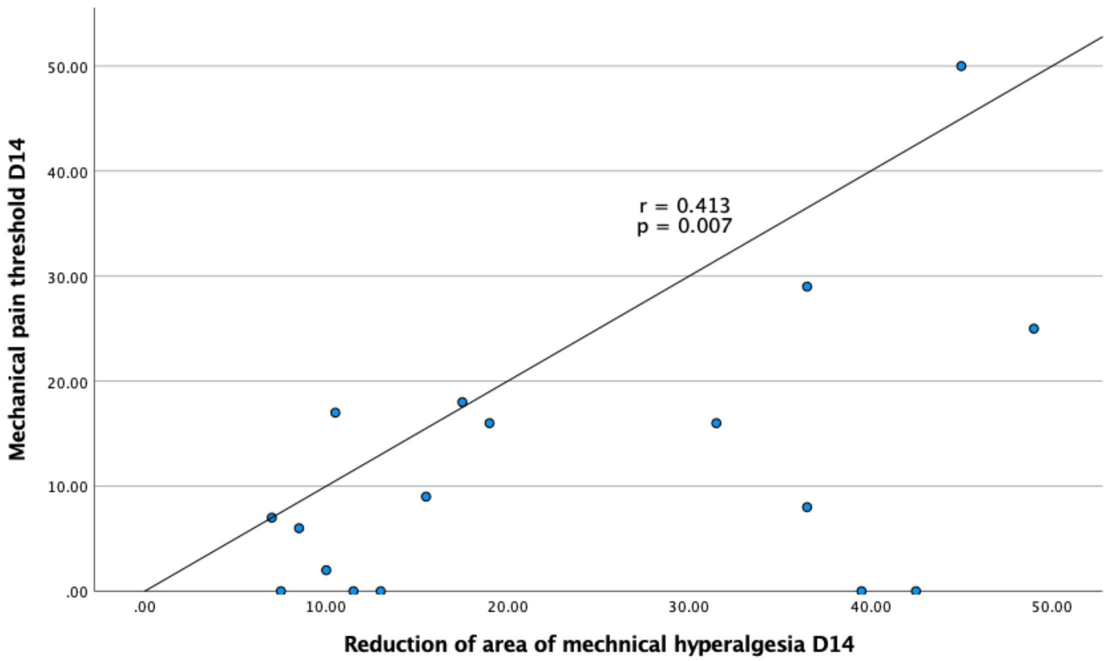

Scatterplot of reduction of the area of mechanical hyperalgesia (cm^2) and correlation with decreasing mechanical pain threshold at D14 (r = 0.413, p = 0.007).
